# Survivors’ perceptions regarding the follow-up of pain complaints after breast cancer treatment: Distinct coping patterns

**DOI:** 10.3389/fpsyg.2022.1063705

**Published:** 2023-01-12

**Authors:** Yaël Slaghmuylder, Emelien Lauwerier, Peter Pype

**Affiliations:** ^1^InterProfessional Collaboration in Education, Research and Practice (IPC-ERP), Department of Public Health and Primary Care, Faculty of Medicine and Health Sciences, Ghent University, Ghent, Belgium; ^2^Department of Experimental-Clinical and Health Psychology, Faculty of Psychology and Educational Sciences, Ghent University, Ghent, Belgium; ^3^Department of Public Health and Primary Care, Faculty of Medicine and Health Sciences, Ghent University, Ghent, Belgium

**Keywords:** breast cancer survivors, chronic pain, coping, beliefs, qualitative analysis

## Abstract

**Introduction:**

After finishing cancer treatment, breast cancer survivors often experience both physical and psychosocial symptoms such as pain. In some, pain can persist for months or even years. Pain is a complex experience. Its occurrence and maintenance are explained through interactions between multiple factors, which are biological/physiological, psychological, and social in nature. Unaddressed needs related to this problem - such as insufficient pain relief, limited validation of the problem, and minimal physical and psychological support - may cause severe disability and negatively impact well-being and quality of life. This study investigated how breast cancer survivors perceive their (chronic) pain complaints to be addressed during follow-up care. Furthermore, we explored how they coped with the way their trajectories happened to unfold.

**Methods:**

We conducted four focus groups with a total of thirty-one breast cancer survivors. Each focus group consisted of an asynchronous part with an online discussion platform and a synchronous part through video calls. Data analysis was guided by the Qualitative Analysis Guide of Leuven.

**Results:**

Narratives revealed the unmet needs of survivors and showed variability in the lived experiences of having to deal with pain. Some survivors tend to ignore the pain, while others look for solutions to reduce pain. A third coping pattern is accepting pain and its impact. Furthermore, how survivors cope with pain is influenced by intrapersonal, interpersonal, and societal processes. For example, pain-related beliefs and prejudices among healthcare providers, family, friends, colleagues, other cancer survivors, and society could possibly steer a survivor towards a certain way of coping. In these processes, the role of healthcare providers seems pivotal. For instance, when survivors do not feel heard or taken seriously by healthcare providers, their acceptance of pain can be impeded.

**Discussion:**

To conclude, a person’s way of coping with pain and the associated needs is dynamic and influenced by factors at multiple levels such as the intrapersonal, interpersonal and societal level. To sufficiently address the problem of pain among cancer survivors, we therefore also need actions that tackle the health care system and its stakeholders, as well as the public debate concerning cancer follow-up care.

## 1. Introduction

As early detection and effective treatments of cancer have improved survival rates, the quality of cancer survival has become a topic of interest amongst healthcare providers (HCP) and researchers (Zebrack, 2000). A cancer survivor is a person with a history of cancer that is beyond the acute diagnosis and treatment phase (Leysen et al., 2017). One of the largest subgroups amongst cancer survivors are breast cancer survivors (BCS) (Stichting tegen Kanker, 2021). BCSs often experience both physical and psychosocial symptoms in the aftermath of cancer treatment (Cooney et al., 2013; McFarland et al., 2018). One of the most common symptoms is pain (Runowicz et al., 2016). About 13%–51% of BCSs report pain complaints. In some, these complaints may become chronic and result in severe disability and lower levels of well-being and quality of life (Runowicz et al., 2016; Neefs, 2017; McFarland et al., 2018).

Multiple factors (i.e. biomedical, psychological, and social factors) can play an influential role in the pain experience and related disability (De Ruddere et al., 2014). Exemplary factors are mood, depression, anxiety, stress, cognitions, way of coping, self-image, sexuality, sleep, fatigue, physical functioning, social interactions, functioning at work, and financial insecurities (Seber et al., 2016; Tait et al., 2018; Armoogum et al., 2020; Fekrmandi et al., 2020). Some of these factors also play a pivotal role in the persistence of pain complaints (Lotsch et al., 2018). For instance, when survivors adopt a helpless orientation towards pain, the risk of enduring pain is higher in comparison to more active ways of coping (Burton et al., 2007; Karademas et al., 2007; Haanpaa et al., 2011; Tait et al., 2018). Different ways of coping with persistent pain can be distinguished amongst BCSs such as altering daily activities to work around the pain, acceptance of pain as a normal part of cancer recovery, and social comparison as a means to dismiss concerns or to take comfort when others’ complaints seem more severe (Armoogum et al., 2020).

Despite the acknowledged impact of pain in the literature, pain is often not timely assessed nor adequately monitored in practice (Neefs and Lauwers, 2016; Runowicz et al., 2016; Slaghmuylder et al., 2022). Cancer survivors report a lack of physical and psychological support after the initial treatment phase (Neefs, 2017). They feel abandoned by their environment (Neefs and Lauwers, 2016). For example, they experience reduced contact with the medical staff and the loss of their safety net such as family and friends (Charlier et al., 2012; Kang et al., 2020). Their environment often expects they can resume their life as it was before the cancer diagnosis. Others’ expectations about illness, cancer treatment, and social relationships can influence the emotions and behaviour of a cancer survivor (Zebrack, 2000). For example, when BCSs with chronic pain cannot meet the social norms regarding remission or cure with return to daily activities, they might experience self-blame and will no longer report their pain complaints (Charlier et al., 2012; Kang et al., 2020). As a result, the follow-up of pain complaints often stays unmet (Aaronson et al., 2014). Unmet needs can worsen over time, giving rise to more distress and complex situations in the long-term (Cooney et al., 2013; Willems et al., 2016; Leysen et al., 2017; Wang et al., 2018).

The purpose of this study was to investigate how BCSs perceive their pain complaints to be addressed during follow-up care. More particularly, we were interested in survivors’ narratives on the extent to which their needs are sufficiently addressed or left unmet. Furthermore, we explored how they coped with the way their trajectories happened to unfold.

## 2. Methods

### 2.1. Study sample

We recruited BCSs with the help of peer support groups for cancer patients, a national breast cancer organisation (i.e. Think Pink), social media platforms, primary HCPs, and HCPs working in breast clinics in Flanders, Belgium. The target population were BCSs with acute or chronic pain complaints. Inclusion was based on a breast cancer diagnosis in the past years (the number of years was not limited), a completed cancer treatment (without imposing a specific time period), and perceived pain complaints. An exception was made for survivors receiving adjuvant endocrine therapy. To prevent secondary or contralateral breast cancer, some BCSs follow endocrine therapy for a period of 5–10 years after completing their cancer treatment (Burstein et al., 2019). Because musculoskeletal pain is a well-known side-effect of endocrine therapy, we decided to include this population (Seber et al., 2016). Patients with a (self-reported) life prognosis of fewer than 6 months or a psychiatric illness that required primary treatment and follow-up, were excluded. Purposeful sampling was applied based on maximum variation according to current pain intensity and pain duration. As such, we included participants whose pain intensity rating and/or duration of the pain complaints deviated from the survivors who were already participating.

### 2.2. Ethics

The study was approved by an independent Committee for Medical Ethics affiliated with Ghent University Hospital (reference number BC-09130). The participants signed an informed consent after receiving a written explanation of the nature, purpose, and duration of this research.

### 2.3. Data collection

Focus groups were conducted from February 2021 to March 2021. We organised these focus groups online. At the time of data collection, physical contact had to be limited given the COVID-19 pandemic. Additionally, face-to-face focus groups are time and place-bound and make it more difficult to reach certain populations (Janghorban et al., 2014; Stewart and Shamdasani, 2017).

Prior to the focus groups, participants completed a short questionnaire by email to collect demographic as well as health data such as the cancer diagnosis, followed treatment, pain intensity, and pain duration (Ayala and Elder, 2011; Victorson et al., 2019). Each focus group consisted of two parts. First, an asynchronous part was organised with the online discussion platform Focus Group It, lasting 11 days.^1^ During the first 8 days, the moderator asked a new question each day to explore BCSs’ perceptions regarding follow-up of (chronic) pain complaints. The participants got the opportunity to answer each question without specific requirements (such as word count), read the reactions from other participants, and start a conversation with others. During the last 3 days, the moderator probed with a few more in-depth questions (Lijadi and van Schalkwyk, 2015; Stewart and Shamdasani, 2017). We opted for an introductory asynchronous part because it can preserve anonymity whilst discussing sensitive subjects. In addition, the chance of socially desirable answers is reduced (Stewart and Shamdasani, 2017; Reisner et al., 2018). A semi-structured topic guide was used (Supplementary File 1). Relevant topics were identified based on existing literature and questionnaires (Ostelo et al., 2003; Taylor et al., 2013; Huijg et al., 2014; Marlow and Wardle, 2014; Atkins et al., 2017; Chekuri et al., 2018; Scott et al., 2019). For example, the literature suggests a step-wise care model for managing chronic problems to efficiently allocate resources by offering care to all, but the most intensive care only to those patients in highest need (Von Korff and Tiemens, 2000). A description of stepped care was provided and the usefulness of such a model was discussed, keeping in mind their own situation. This description was based on existing literature and no confusion regarding this description was noted. Finally, throughout the data collection process, the topic guide was iteratively adapted according to new insights from our focus groups (Reisner et al., 2018).

Second, a synchronous part took place with the video conferencing platform Microsoft Teams (Janghorban et al., 2014). During these video calls, the major themes from the asynchronous part were discussed in more detail, the answers given were further explored, and group discussion was encouraged (Ayala and Elder, 2011). The synchronous focus groups were recorded and the video records were transcribed verbatim for analysis. Non-verbal aspects were also noted as an aid in interpreting the data (Silverman, 2011).

### 2.4. Data analysis

Sampling, data collection, and analysis were iterative processes (Draucker et al., 2007; Kohn and Christiaens, 2012; Foley and Timonen, 2015). Sample characteristics were analysed using descriptive statistics in the software program IBM SPSS Statistics 26 (SPSS Corporation, Chicago, IL).

The Qualitative Analysis Guide of Leuven (QUAGOL) was used as a guideline in the analysis of the qualitative data (Dierckx de Casterle et al., 2012). This guide was inspired by the constant comparison method, also applied in Grounded Theory. The proposed method of analysis within this guide was adapted to the current research design (Draucker et al., 2007; Kohn and Christiaens, 2012; Foley and Timonen, 2015), consisting of eight steps. (1) Transcripts of the focus groups were thoroughly read several times to familiarise with the data and a narrative report was drawn up for each focus group. These reports provide a narrative description of the essence and key storylines of the focus group in answer to the research questions. (2) The first focus group was independently reviewed by the three authors (YS, EL, PP), with relevant data being clustered into concepts. Concrete experiences were replaced by concepts and were presented in a scheme. (3) The obtained conceptual schemes were compared and their suitability was verified: do these concepts reflect the research questions and can these concepts be linked to the data? One common list of concepts was drawn up without imposing a hierarchical order. This non-hierarchical list of concepts was entered into the Nvivo software program (Dierckx de Casterle et al., 2012; Atkins et al., 2017). (4) The first focus group was again individually reviewed by two authors (YS, EL) and relevant fragments were linked to concepts. If necessary, existing concepts were adapted, new concepts were added, or concepts were split into several sub-concepts. A list of concepts and associated statements was obtained inductively. (5) The lists of the two authors were compared and discussed until a consensus was reached. The third author (PP) was also involved in this comparison to reflect on the coding process. (6) One author (YS) individually determined the concepts of the other three focus groups and linked specific fragments to concepts. These concepts were again discussed within the research team (YS, EL, PP). (7) One list of concepts was ultimately obtained. The concepts were then grouped and different clusters were defined and again discussed within the research team (YS, EL, PP) until consensus was reached. Identification of the different clusters was inspired by the Theoretical Domains Framework (Cane et al., 2012; Atkins et al., 2017) and a model of injustice domains in pain (Mathur et al., 2022). The former comprises cognitive, affective, social, and environmental influences on behaviour and behaviour change (Cane et al., 2012; Atkins et al., 2017). The latter describes interacting interpersonal, structural, and cultural injustice domains that influence intrapersonal processes and contribute to pain outcomes (Mathur et al., 2022). (8) Finally, a synthesis of the study results was discussed within the steering group of our overall research project. As this group involves a diversity of members such as academic scholars, professionals in the field, and patient representatives, this may be regarded as an important step in peer reviewing the authors’ interpretations and therefore adds to the validity of the results.

**Figure 1:**
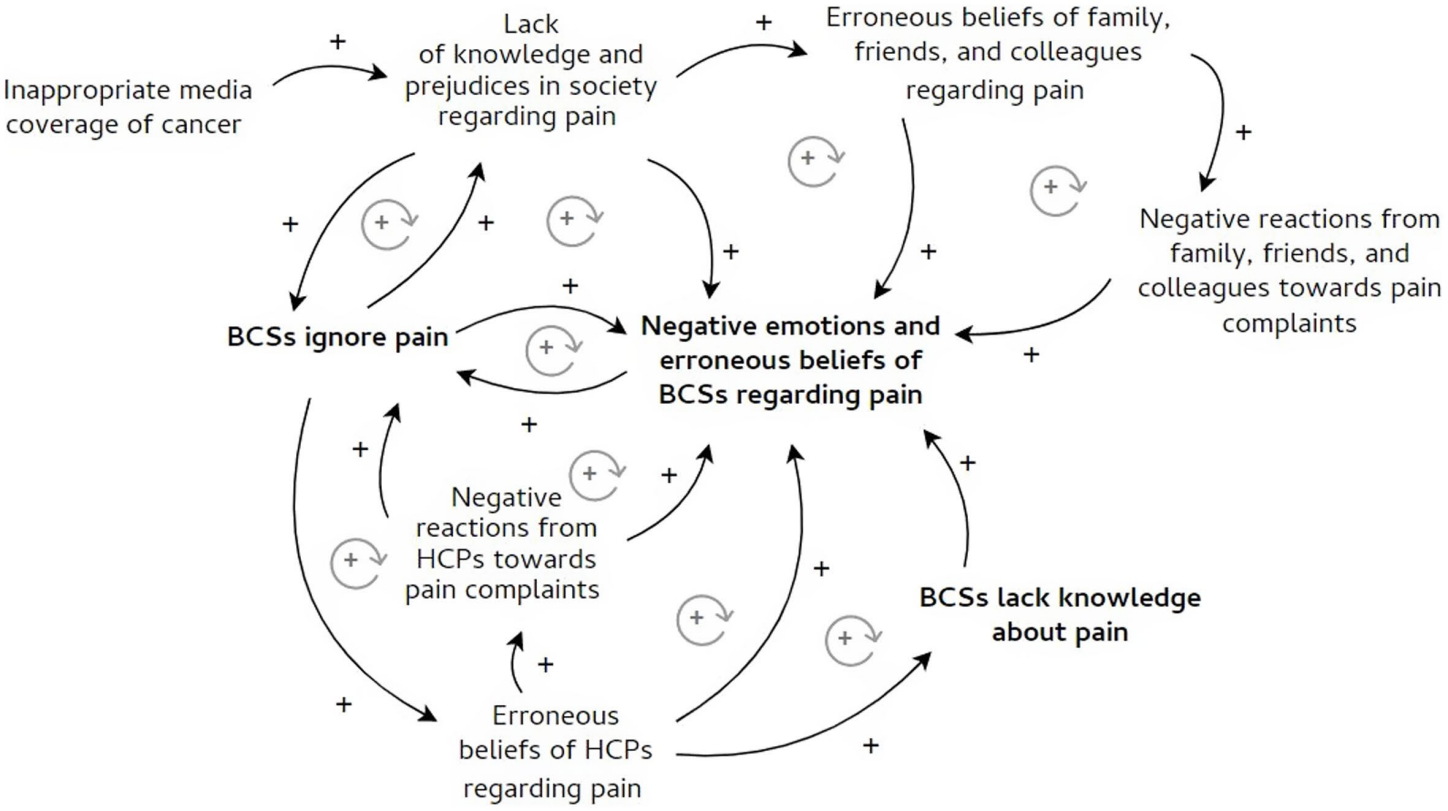
Ignoring pain and influencing processes. Figure shows the pattern of ignoring pain and its influencing processes. The processes are located on an intrapersonal, interpersonal, and societal level. The intrapersonal processes are shown in bold. The influencing relationships between processes were translated into words-and-arrows diagrams. The positive arrows represent relationships that reinforce the pattern of ignoring pain. The negative arrows stand for inhibiting relationships. When a relationship is reciprocal, a circular arrow in grey was added. The following abbreviations were used: BCS, breast cancer survivor; HCP, healthcare provider.

**Figure 2:**
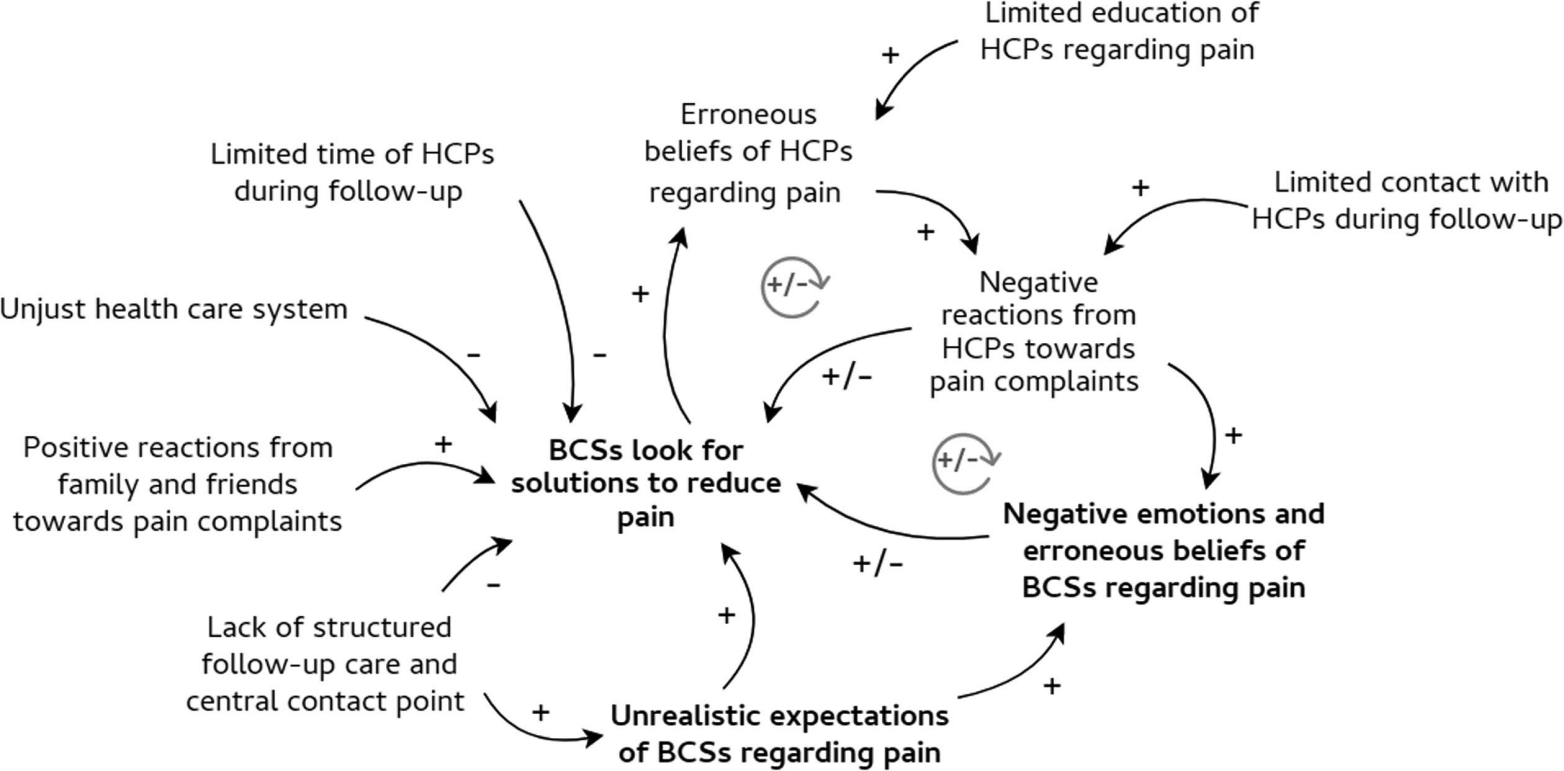
Solving pain and influencing processes. Figure shows the pattern of solving pain and its influencing processes, i.e. intrapersonal, interpersonal, and societal processes. The intrapersonal processes are shown in bold. The influencing relationships between processes were translated into words-and-arrows diagrams. The positive arrows represent relationships that reinforce problem-solving behaviour in breast cancer survivors. The negative arrows stand for inhibiting relationships. When a relationship is reciprocal, a circular arrow in grey was added. The following abbreviations were used: BCS, breast cancer survivor; HCP, healthcare provider.

**Figure 3:**
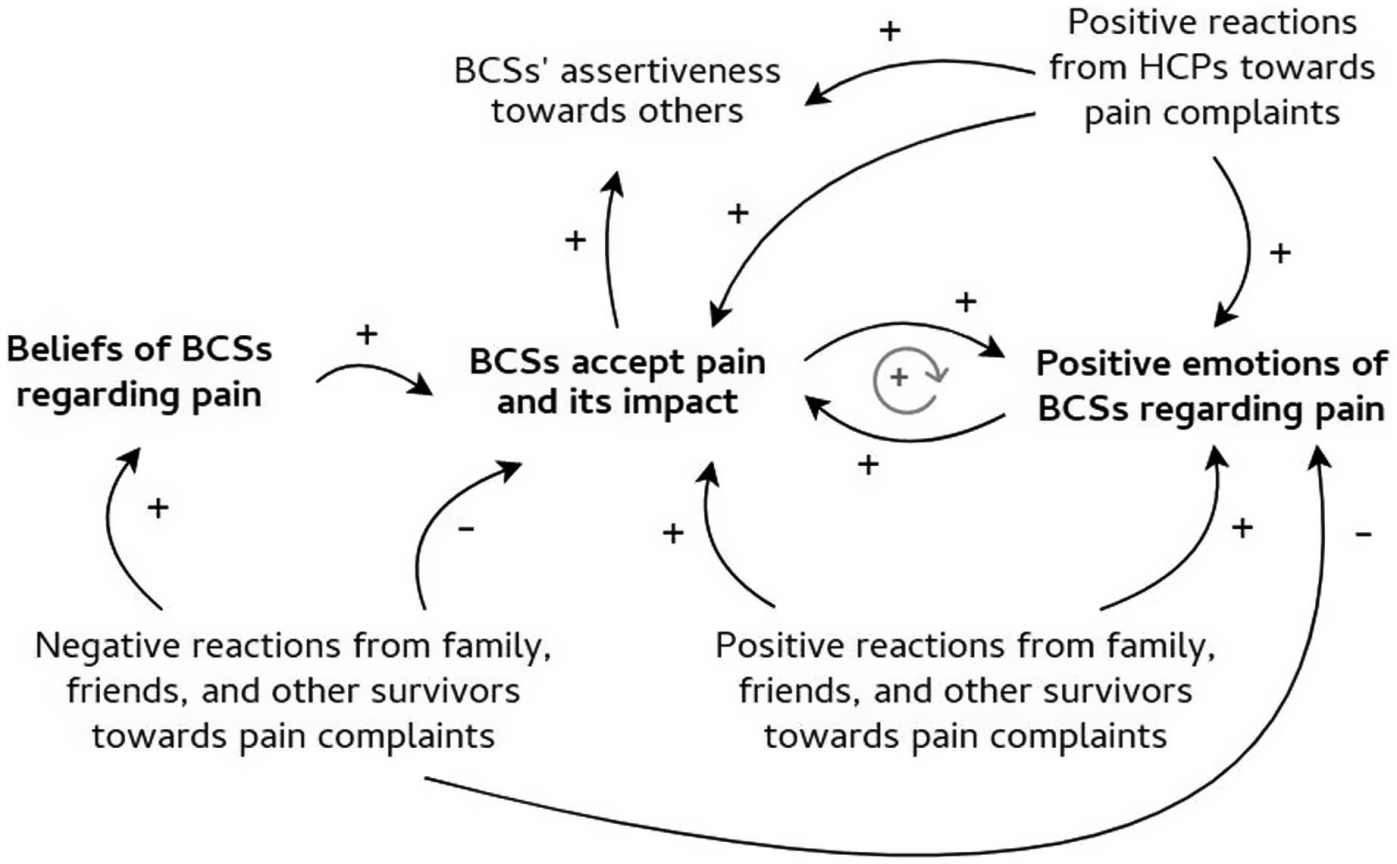
Accepting pain and influencing processes. Figure shows the pattern of accepting pain and its influencing processes on an intrapersonal, interpersonal, and societal level. The intrapersonal processes are shown in bold. The influencing relationships between processes were translated into words-and-arrows diagrams. The positive arrows represent relationships that reinforce the acceptance of pain, whilst the negative arrows stand for inhibiting influences. When a relationship is reciprocal, a circular arrow in grey was added. The following abbreviations were used: BCS, breast cancer survivor; HCP, healthcare provider.

We described three different ways of coping with pain and related processes at an intrapersonal, interpersonal, and societal level, as well as cross-level relationships. To visualize the most prominent relationships, we created causal loop diagrams (Figures 1–3). First, the intrapersonal processes were identified and situated at the centre of the diagrams, shown in bold. Second, relationships between concepts at the interpersonal and societal level were described and presented around the concepts at the intrapersonal level. These relationships were translated into words-and-arrows diagrams. When a causal link demonstrated a reciprocal relationship, a circular arrow in grey was added to show the feedback loop. These feedback loops represent relationships and their polarity. A positive polarity (+) stands for a reinforcing relationship and a negative polarity (−) stands for an inhibiting relationship. If all the arrows in the loop are positive or there is an even number of negative arrows, the loop is reinforcing (+). In this study, the polarity shows whether the loop has a reinforcing or inhibiting influence on a certain coping pattern (Baugh Littlejohns et al., 2018). Finally, significant quotes were added when relevant (Dierckx de Casterle et al., 2012). Quotes from the asynchronous focus groups were referred to as (AS) and from the synchronous focus groups as (S).

### 2.5. Reliability

The trustworthiness of the data was increased through investigator triangulation. The team approach increased the ability to get to the essence of the data and correct misunderstandings. It provided an in-depth and rich understanding of the research phenomenon. Furthermore, each team member represents a different discipline (i.e. a general practitioner, a psychologist, and a speech-language therapist) which contributed to the quality of the discussions, the integration of different perspectives, and the reliability of the results (Dierckx de Casterle et al., 2012).

## 3. Results

### 3.1. Study sample

Four focus groups were organised. Each group consisted of seven to eight participants with a total of 31 participating BCSs. The pain was labelled ‘chronic’ when persisting longer than 3 months. Therefore, four participants had acute pain and 27 participants had chronic pain. The sample characteristics are summarized in Table 1.

**Table 1:** Sample characteristics.

Sample characteristics		
Age in years, mean ± SD		51 ± 7
Sex, *n*	Female	30
	Male	1
Country of birth, *n* (%)	Belgium	30 (96.8)
	Other	1 (3.2)
Knowledge of Dutch, *n* (%)	Very good	30 (96.8)
	Good	1 (3.2)
Education, *n* (%)	High school	9 (29.1)
	Bachelor’s degree	17 (54.8)
	Master’s degree	5 (16.1)
Working, *n* (%)		18 (58.1)
Adjuvant hormone therapy, *n* (%)		17 (54.8)
Pain severity ranged 0–10, mean ± SD min–max		4.8 ± 2.5 1–8.5
Pain duration in months, mean ± SD min–max		36 ± 45 2–228

Summarizes the characteristics of our study sample of 31 breast cancer survivors. The following abbreviations were used: SD, standard deviation; *n*, absolute frequency; %, relative frequency; min, minimum; max, maximum.

The asynchronous focus groups consisted of 756 responses in total. The majority of participants answered every question with an average of 24 responses per participant (minimum 11, maximum 54). The video calls of the synchronous focus groups had a mean duration of ~2 h. Similar themes were discussed during the two parts, but some themes were explored in more depth during the synchronous part.

### 3.2. Coping with pain in relation to intrapersonal, interpersonal, and societal processes

BCSs experience different kinds of pain complaints with an impact on their mood, self-image, self-confidence, daily functioning, quality of life, and financial security. They want to be prepared for the side effects of treatment and informed about possible pain problems. They expect their follow-up care to start soon enough, to last long enough, to be individualised, and to be integrated. In addition to this, they want others to understand their pain complaints and feel useful again.

However, follow-up of pain complaints often stays unmet. Many BCSs feel unprepared to deal with pain after cancer treatment. They also do not always feel supported in their pain complaints by others. Pain experiences are highly individual and difficult to predict, as is how pain is dealt with. In this study, we identified three different patterns of coping with pain during breast cancer follow-up. Furthermore, coping is influenced by processes on an intrapersonal level, as well as on an interpersonal and societal level. The ways of coping with pain and their relationships were described below and visualised with causal loop diagrams. The intrapersonal processes are shown in bold (Figures 1–3).

#### 3.2.1. A pattern of ignoring pain

Figure 1 shows the intrapersonal, interpersonal, and societal processes that influence the pattern of ignoring pain.

##### 3.2.1.1. Ignoring pain and intrapersonal processes

Some of the participating BCSs ignore their pain experience or seek distractions as a coping mechanism for pain. They will not talk about pain complaints, nor will they seek support. Different intrapersonal processes influence this pattern. Namely, ignoring pain can be influenced by one’s own emotions, expectations, and beliefs.

Some BCSs state that they focus on returning to their ‘normal’ life as quickly as possible. They do not want to be confronted with the impact of pain on their functioning. They continue their lives as before without guarding their new boundaries.

“I try to pretend that everything is ‘normal’ again. You do that for yourself too. You convince yourself every day that it is already much better than before.” (AS)

A belief that is prevalent amongst some BCSs, is the assumption that their pain experiences are not cancer-related and are caused by menopause or ageing. These BCSs often do not know the cause of their pain experiences. As a result, they endure the pain and do not expect any pain reduction. Furthermore, they do not talk about pain but rather refer to ‘discomfort’.

“For the discomfort in my arm - I do not like to call it pain - I do not have much help right now. I also do not think the pain can be improved.” (AS)

Finally, a few BCSs report doubts about the credibility of their own pain complaints. Therefore, they feel guilty because they might use health resources without deserving them. These feelings can result in ignoring pain and not asking for support in the future.

“During the rehabilitation programme, I felt a bit guilty because I might occupy a spot that I was not entitled to. (The spots for the rehabilitation programme were limited.)” (AS)

##### 3.2.1.2. Interpersonal and societal influences

The above-mentioned intrapersonal processes are influenced by past interactions with HCPs. For example, many of the participating BCSs do not feel heard or taken seriously because of HCPs’ reactions to pain complaints. They experience that some HCPs have no explanation for the pain, ignore or postpone answering questions about pain, do not give any information about pain, do not actively search for solutions, and pass on their responsibilities to other HCPs. Furthermore, they sometimes suggest that pain is easily cured and that the patient should put in more effort or have more patience. Because of this struggle to be heard, BCSs feel belittled, hurt, misunderstood, frustrated, angry, sad, powerless, helpless, desperate, or exhausted. As a result, BCSs’ beliefs are reinforced regarding pain complaints not being cancer-related or not being able to reduce pain.

“Due to a previous disappointment - professionals not knowing how to deal with severe pain - I did not contact my health care providers again.” (AS)

Participating BCSs also state that some HCPs hold erroneous beliefs about pain and – as a result – do not always believe the pain complaints. For example, when BCSs do not want to take medication to reduce pain, when BCSs are still able to be active despite the pain, and when pain complaints appear atypical in the eyes of HCPs. As a result, several participating BCSs believe that their pain complaints are not common or even not real. Moreover, feelings of doubt and guilt are triggered.

“When healthcare providers did not react to my severe pain complaints, I felt desperate, helpless, sad, and misunderstood. I even felt 'different'. Do other women not suffer from pain? Was I a fraud, a weakling?” (AS)

The behaviour of family and friends in interaction with the survivor also influences intrapersonal processes. If a BCS looks good again (e.g. their hair has grown back), the environment expects the survivor continues life as before the diagnosis. Family and friends mitigate the pain or believe the pain complaints are exaggerated. Some BCSs also feel avoided by family and friends. They sense that their environment is afraid to talk about pain and associated emotions. As a result, a few of the participating BCSs fear that their environment will not consider them as a whole person or will not love them because of their pain complaints. Others believe their environment cannot help them with their pain. Therefore, BCSs do not discuss pain with their family and friends and minimise the pain. Furthermore, some BCSs state that they prioritize their environment before themselves (e.g. they do not hand over household tasks despite the pain), because they do not want to burden their family and friends or make them worried.

“My environment does not handle pain after treatment well. I feel that when I say I am in pain, they either minimize it or think I am exaggerating. As a result, I do not always say that I am suffering. But this starts to impact my mental well-being. I have to constantly tell myself 'do not complain' and that triggers even more pain.” (AS)

Interactions within a work context also influence the intrapersonal processes of BCSs. Some BCSs mention feeling supported by colleagues when returning to work, others did not. Colleagues often expect the BCS to act the same as before the diagnosis. As a result, BCSs feel misunderstood, angry, a simulant, and afraid of being perceived as less productive employees. They try to act ‘normal’ and will therefore not share their pain complaints during work, try to ignore the pain, and take pain medication more quickly.

“I am dressed decently, put on a little make-up and my colleagues say 'wow, you look good'. In their eyes, everything is okay. But they only look at the appearance. They do not know how I really feel. I find that very frustrating. I cannot explain it to them, because they say 'pain, what is pain, take acetaminophen’. But it is not that simple. It hurts a lot more than that pain alone. It's also mental pain.” (S)

On a societal level, the participating BCSs perceive a lack of knowledge on the part of society regarding pain after cancer treatment. They feel that chronic pain and associated emotions are still taboo for a lot of people. Furthermore, society sometimes imposes shame on survivors. For example, others find it inappropriate when they react with humour to their problems or when they do not hide their changed body. In accordance with the interpersonal level, society also holds certain prejudices regarding pain after cancer treatment. For example, society presumes that pain is less severe when the patient did not receive chemotherapy or radiotherapy. These prejudices can alter the beliefs of BCSs (e.g. the pain is not real) and prompt certain behaviour (e.g. pursuing a ‘normal’ life).

“On the outside, people could not see anything. I did not get chemotherapy, so there was no hair loss. As a result, some people and colleagues made me feel like I had only 'half' a cancer. In their opinion, I should not complain too much because it could have been much worse. No chemotherapy, no radiation, so it is not that bad, right?” (AS)

Several participating BCSs believe that these prejudices are stimulated by media reports because these only focus on positive stories about being cured without attention to possible side effects. They also feel that the media holds the patient responsible for their cancer diagnosis. These prejudices act as a barrier to talking about pain and force survivors to ignore pain.

“The media represents a ‘good news show’ regarding cancer. We see many ‘brave’ patients who gratefully cycle up Mont Ventoux or climb Mount Everest after they are ‘cured’. This distorts the perception of society. The media is full of advice about healthy eating and exercise. It is even said that you will not get terrible diseases 'as long as you live healthily'. In other words, cancer patients are probably partly responsible themselves.” (AS)

The participating male BCS states that society also often sees breast cancer as a disease that only affects women. Furthermore, the impact of breast cancer on male patients is underestimated. As a result, he feels that many male BCSs deny their diagnosis and any side effects they might experience afterward, imposing a threshold to seeking support. This threshold is also reinforced by female support groups that do not always welcome a male BCS.

“Many men will not share their diagnosis with others. They will no longer go to the pool in their swimming trunks. Many men hide themselves. And that's a shame. Because then support might not be available when they need it.” (S)

#### 3.2.2. A pattern of solving pain

Figure 2 shows the intrapersonal, interpersonal, and societal processes that influence the pattern of solving pain.

##### 3.2.2.1. Solving pain and intrapersonal processes

Some of the participating BCSs actively look for solutions with the aim to reduce pain. For example, they attempt to understand the pain better by asking HCPs for information or searching for information themselves, they try out different pharmacological treatments, they take initiative to contact HCPs outside the hospital, or they seek out more non-conventional treatments (e.g. curcumin supplements, neural therapy, and acupuncture). These BCSs acknowledge that their pain complaints are linked to cancer treatment, and should not be seen as normal symptoms of ageing.

“I do not want to accept the pain I am experiencing right now. I want to keep looking for solutions, medication, therapies… that can provide improvement, pain relief. The way I feel right now is not okay. Something has to be done to make it better. I really hope that is possible.” (AS)

However, some BCSs state that they did underestimate the severity of possible pain problems in advance. When BCSs experience that their functioning is more limited due to pain complaints as expected, they feel frustrated and angry. These feelings can fuel their motivation to seek solutions for pain reduction.

“After my treatment, the pain in my left arm was less severe than it is now. Since October last year, I suddenly felt that pain again. I was really in panic and caught by surprise because I thought the worst was through. Apparently, that is something I cannot think of anymore because it can flare up at any time. That was very intense for me.” (S)

BCSs – who actively try to solve pain problems – are often convinced or expect that pain can be reduced. Especially survivors whose treatment did not end that long ago, want to fight and improve their functioning.

“In the first place, I would prefer that the pain disappears, which is why I seek help. I also want to go back to work, preferably pain-free. I know it is also a bit of acceptance. But there are many pieces of acceptance, which makes it difficult and confrontational sometimes.” (AS)

When HCPs cannot offer a solution to reduce their pain, they feel abandoned, disappointed, frustrated, and alone. In contrast to this, several participating BCSs believe it is their own responsibility to talk about pain and seek solutions for their pain complaints. They do not expect medical specialists to ask about pain or give information. When they lack care, they blame themselves as stated in the quote below.

“To be honest, I do not get any care for my pain complaints. I live on painkillers. Maybe, this is my own fault. Because after all these years, I gave up on repeating my pain complaints over and over again.” (AS)

Finally, a BCS’s aim to solve pain can be altered by certain beliefs. For example, a few of the participating BCSs are convinced that pain is related to one’s personality. They belief that other survivors fake their pain or complain too much. Therefore, they will not always talk about their own problems or seek support to solve the pain, because they do not want to appear as a complainer or exaggerator.

“Pain is neither measurable nor visible. There will be people who spend their whole lives 'faking' all sorts of ailments and pain complaints in order to be lazy, in order to benefit from the system. When physicians do not know you thoroughly, they cannot know if you are presenting symptoms worse than they really are.” (AS)

##### 3.2.2.2. Interpersonal and societal influences

Another influencing factor that might reduce problem-solving behaviour in BCSs, is the lack of a central contact point amongst HCPs. BCSs mention they often do not know who to turn to when they have questions or want to look for solutions. Furthermore, some BCSs are not monitored by a permanent team of HCPs. They do not have much contact with the same medical specialists. Additionally, BCSs feel that the time of medical specialists during follow-up consultations is limited, making it more difficult to talk about side effects such as pain and collaborate on possible solutions.

“Experiencing a threshold to talk about pain, might also be related to the fact that an appointment only lasts ten minutes. If you think ‘I will be out of here in five or ten minutes’, then you are also less eager to take initiative and ask questions.” (S)

BCSs also experience that some HCPs normalise the pain problems too much. They feel misunderstood when HCPs indicate that pain problems do not impact their functioning to a great extent.

“In the breast clinic, we were urged to go back to work as soon as possible. They said ‘That is good for your mental well-being and will make you belong again’. But they keep quiet about the fact that at the same time endocrine therapy drags you into the deep. In other words, you may be ill for a while, but afterward 'back to normal' as soon as possible.” (AS)

Furthermore, not every hospital has the same resources such as rehabilitation programs or outpatient consultations. Still, referral to other disciplines outside the hospital often does not happen or happens too late. Additionally, the added value of non-medical disciplines is not always recognised. Participating BCSs believe that the biomedical approach of some medical specialists is a result of education. Their training focuses too much on curing cancer and avoiding cancer recurrence with little regard for side effects. BCSs feel that – as a result – some HCPs do not know how to manage chronic pain or do not dare to start a conversation about pain. It empowers BCSs to keep talking about their pain complaints until they feel heard and supported by HCPs.

“Pain and discomfort remain difficult to discuss. Some phrases that are in every training of medical professionals: ‘it will pass’, ‘we are not going to complain too much, are we’, ‘other patients do not have that though’, and ‘you should be happy that you are still alive’.” (AS)

Finally, some BCSs feel the health care system is unjust. For example, there is no adjusted care available for male breast cancer patients, reimbursement for breast protheses or lymphatic drainage is limited, or administrative procedures for receiving funding for informal care are not adapted to the specific needs of BCSs. These structural factors complicate BCSs’ struggle to beat pain.

“The first year after my surgery, I was able to follow lymphatic drainage with reimbursement. I was treated by a physiotherapist. The treatment hurt, but also felt good, and was beneficial. Now it is no longer refundable. If you do not need lymphatic drainage, you will not go to a physiotherapist for it. So, what is the problem? The fact that oedema can still appear after all the treatments shows that a lot of people suffer from it and would benefit from being reimbursed longer. I think we have suffered enough already!” (AS)

In contrast to the above-mentioned barriers to problem-solving behaviour, BCSs who experience support and understanding from their family and friends feel more secure and empowered to solve pain (e.g. by talking with HCPs and asking for help).

“I think it is in my nature to search for appropriate care when I experience pain, but support from my environment - usually my husband - helps me over the threshold when in doubt.” (AS)

Next to family and friends, HCPs can also play an important part in encouraging BCSs to take initiative and look for appropriate support. When HCPs acknowledge the pain and search – together with the survivor – for possible solutions, the participating BCSs often trust their HCP more. Another contributing factor to trust is reassurance. When HCPs reassure BCSs that pain complaints are often prevalent after finishing cancer treatment and are not always a sign of cancer recurrence, BCSs have more faith in their HCP. Trust can lower the threshold for discussing pain and asking for help. Additionally, some BCSs feel encouraged and motivated again to attempt solving their pain problems.

“If the healthcare provider is understanding, it makes me feel seen and gives reassurance. It gives me the courage to move on. The mind is very strong and can positively influence pain. But, therefore, you first have to be positively encouraged.” (AS)

#### 3.2.3. A pattern of accepting pain

Figure 3 shows the intrapersonal, interpersonal, and societal processes that influence the pattern of accepting pain.

##### 3.2.3.1. Accepting pain and intrapersonal processes

Some of the participating BCSs seem to accept pain problems and their impact. They adjust their goals in life, try to dose activities, and set up new boundaries. Accepting pain can also increase their self-worth and assertiveness towards others. They talk about their pain experiences with HCPs with whom they have a trusting relationship, with fellow BCSs, and with family or friends. Some BCSs also state that – as a result of acceptance – they no longer need external help. They focus on self-care, mindfulness, and meditation. In our study sample, BCSs with chronic pain were often more inclined to accept their pain complaints. The participating BCSs – who more recently finished their cancer treatment – were still searching for a way to accept pain and manage their new boundaries.

“Learning to live with pain means that now - after ten years - I know that I have done everything within my power to get to the level where I am today. Would I like to be stronger, completely fear and pain-free? Of course. But I have tried so much and put so much energy into going to the physician whenever I felt pain, that I stopped going.” (AS)

When BCSs think that other survivors have more pain complaints, accepting pain becomes easier. These survivors are often grateful, count themselves lucky, and try to focus on what is still possible.

“I also experience pain, but that's all relative. I do think there are people who have a lot more pain complaints than me.” (S)

Furthermore, some BCSs are convinced pain is part of survivorship and cannot be solved. Especially postoperative pain is more easily accepted because it is not considered chronic pain but a part of the healing process. When HCPs cannot provide a solution for the pain, they are still satisfied with the offered support. However, it is important to notice that in our study this pattern mainly occurred amongst survivors with acute pain.

“My thoughts about pain … At the moment, I often think ‘pain must be normal’, because my surgery was not that long ago.” (S)

##### 3.2.3.2. Interpersonal and societal influences

The behaviour of HCPs can reinforce acceptance and certain emotions of BCSs. For example, when HCPs listen to pain complaints, acknowledge the pain, anticipate by actively asking about possible pain complaints, offer information and education about pain, search for different solutions such as non-pharmacological interventions, refer to colleagues when needed, and are open and honest in their communication towards the patient. As a result, BCSs state that they feel understood, reassured, relieved, hopeful, and in control. BCSs often do not expect HCPs to have a ready-made solution but do expect acknowledgement of their pain complaints. Additionally, feeling supported, reduces frustrations and anger when experiencing pain after treatment and facilitates pain acceptance.

“Even though there was no solution for my pain, I could always go to the breast clinic. This made me feel recognized and understood. They will not necessarily take all the pain away, but that support is often enough to have some energy again to start the next day.” (S)

The participating BCSs also feel supported and understood by family and friends. For example, when the environment notices that the BCS is in pain, when they sincerely ask how they are feeling, when they offer practical support, when they adapt to the altered functioning of the BCS, when they listen to pain complaints, and when they confirm how good the BCS is dealing with pain. Whenever the environment acknowledges their pain, BCSs feel supported, encouraged, and mention a positive influence on their mental wellbeing.

“My family and friends remind me where I come from and that I can already do a lot of things. They remind me that I have time and need to be patient.” (AS)

Additionally, BCSs report that they often feel understood by other survivors. Sharing experiences amongst survivors, makes them feel less alone or abnormal. Other BCSs often recognise pain complaints and offer information. This triggers a positive and hopeful attitude and facilitates acceptance of pain.

“I experienced a lot of support from contact with fellow survivors. Hearing that others experience the same, reduces feeling abnormal. It can also help to put pain - which may always be present - into perspective, to try to deal with pain in a certain way.” (S)

Nevertheless, not every participating BCS feels supported by other survivors. Especially when their complaints are not compatible with those of other survivors, they sometimes experience disbelief.

“My mother - a breast cancer survivor - compares my pain complaints to hers. When my nerve pain was worse than hers had ever been, she did not really hear me. I got the feeling that she did not believe me.” (AS)

In line with this, some BCSs do not always feel supported by their close environment, impeding the acceptance of pain. For example, the environment focuses on the physical aspect of pain and gives unsolicited advice such as taking medication to reduce pain, they do not listen to pain complaints, attribute pain to ageing, and start talking about their own ailments.

“Others do not realize that after the treatment you can still suffer from complaints. I have often heard ‘it is over now, you have to be positive and look ahead’, also from other survivors. I am positive, I do look ahead! But that does not mean it is all over. You cannot explain that over and over again every time.” (AS)

Moreover, pain can negatively influence romantic relationships (e.g. physical touch can sometimes be painful). Some of the participating BCSs are convinced that their partner regards sex as a purely physical act. They believe their partner does not make a distinction between sex and other physical gestures such as a hug or a kiss, resulting in a total absence of intimacy. The impact of pain on the romantic relationship and associated beliefs are not discussed with the partner, which makes acceptance of pain and its impact more difficult.

“A relationship is much more than sex. However, my partner does not dare to come close to me, because - in his eyes - this would indicate sex drive. He thinks that he is sparing me, creating a habit of physical distance which is detrimental to our relationship.” (AS)

## 4. Discussion

We aimed at exploring BCSs’ perceptions on follow-up of pain complaints after finishing cancer treatment. This is important as a systematic and coordinated follow-up approach is lacking for BCSs. Survivors must often deal with pain complaints themselves and seek support on their own initiative (Neefs and Lauwers, 2016; Hadi et al., 2017). However, the way BCSs will deal with pain is hard to predict. By exploring the extent to which BCSs’ needs are sufficiently addressed, we identified three patterns of coping with pain and accompanying needs. Namely, some survivors tend to look for ways to ignore the pain complaints, whilst others focus on finding solutions to reduce pain. A third coping pattern is accepting pain and its impact. Furthermore, we noticed a slight difference in the coping patterns between participating BCSs with acute versus chronic pain. Survivors with acute pain are more inclined to accept pain because they believe pain is temporary and will still resolve on its own. Or they actively want to beat the pain problems and limitations pain imposes. BCSs with chronic pain are also looking for a way to accept pain and its impact on life but do so from a different perspective, i.e. they do not expect the pain to disappear completely.

The concept ‘coping’ refers to the myriad actions people use to deal with stressful situations – such as having to live with chronic pain –, influenced by behaviours, perceptions, and cognitions (Skinner et al., 2003). In line with our study findings, the dual process model differentiates assimilative from accommodative coping. Assimilative coping refers to attempts at removing or controlling pain complaints such as staying committed to life goals as before the diagnosis or engaging in activities aimed at solving pain. Accommodative coping focuses on accepting that pain cannot be solved and adjusting life goals that have become unachievable. As such, this model assumes that perceived or anticipated goal discrepancies can lead to self-regulatory processes, i.e. assimilative and accommodative coping (Brandtstädter and Rothermund, 2002; Lauwerier et al., 2012; Van Damme and Crombez, 2018). Though, our study adds a third pattern of coping, i.e. ignoring the pain. Some BCSs instantly try to return to their former life and ignore the impact of pain on their daily functioning. Others doubt the credibility of their pain complaints or do not consider pain a side-effect of cancer treatment, resulting in disregarding pain complaints. Additionally, some survivors ignore pain complaints because they no longer experience pain as problematic. In this case, BCSs use an emotion-focused strategy to cope with pain and grow towards acceptance of pain.

Many classifications of coping are proposed in the literature (Lashbrook et al., 2018). We have gathered some patterns of coping, but – given the fact that there are possibly over 400 different ways of coping – there undoubtedly exist many more (for an extensive overview, see Skinner et al., 2003). Commonly used distinctions are – amongst others – problem versus emotion-focused coping and active versus passive coping (Skinner et al., 2003; Lashbrook et al., 2018; Van Damme and Crombez, 2018). The former distinguishes behaviour directed at solving the stressful situation from behaviour aimed at changing emotional reactions to the stressful situation (Mehrabi et al., 2015; Lashbrook et al., 2018). The latter refers to actions that bring the individual closer to the stressful situation versus attempts to avoid the stressful situation (Skinner et al., 2003; Kraemer et al., 2011; Lashbrook et al., 2018). Additionally, there is an assumption that some strategies are more beneficial in coping with pain after cancer than others. For instance, active or problem-focused coping strategies are often described as more likely to be adaptive, whereas passive or emotion-focused strategies seem to be rather maladaptive (Lashbrook et al., 2018).

However, our purpose was different in a sense that we aimed to explore the approaches to coping through survivors’ narratives, which relates in large part to a more functional approach to coping. In such an approach, no claim is made about specific coping strategies and whether these are helpful or not in the short or long term. We mainly wanted to give voice to our participants and let them describe in depth how they deal with pain and how they perceived it to have evolved to this point. This aligns with functional accounts of coping in the literature, such as the above mentioned dual process model. Another example is a study of Van Damme et al. (2008) in which three different pathways of coping with pain are distinguished, i.e. persistence, problem-solving, and goal adaptivity. These pathways relate in important ways to the patterns that were identified in this study. The idea is that neither of these are beneficial, but much depends on the context in which these arise. Furthermore, coping strategies are often not mutually exclusive. For example, actions can serve both problem-and emotion-focused coping (Skinner et al., 2003; Van Damme and Crombez, 2018). Related to our study, venting about pain-related complaints to family or friends can reduce feelings of frustration and even stimulate support-seeking behaviour in some participating BCSs. Vice versa, contacting HCPs for support also awakens feelings of hope and courage amongst BCSs.

Previous studies have shown that the personal characteristics of patients act as predictors of how they experience pain and deal with pain complaints (Ramirez-Maestre et al., 2014). We identified that how patients cope with pain does not only depend on the patients themselves but is also determined by their environment, in line with the social communication model of pain (Craig, 2015). Interpersonal (e.g. the way HCPs react towards pain complaints) and societal processes (e.g. prejudices in society) can also influence the pain experience and way of coping. Moreover, it is important to acknowledge that these interpersonal and societal processes are not solely the result of pain but form an interactive whole that characterises pain and coping with pain (Meints and Edwards, 2018). Therefore, a person’s way of coping with pain is dynamic, influenced by intrapersonal as well as interpersonal and societal processes. In a longitudinal study of Tighe et al. (2011), breast cancer patients state that symptom experiences affect the way of coping and their interpersonal relationships, which in turn change during the course of cancer diagnosis and treatment. However, based on our results, we cannot conclude how different ways of coping change and/or develop over time during survivorship (Stroebe and Schut, 2010). Though, we did identify several factors that can influence an individual’s way of coping at a fixed point in time.

A main influencing factor of coping are BCSs’ perceptions of erroneous beliefs and prejudices amongst HCPs, in their close environment, and in society. Studies about beliefs and prejudices tend to focus less on the population of cancer survivors (Pescosolido and Martin, 2015; De Ruddere and Craig, 2016; Yeung et al., 2019; Kang et al., 2020). But parallels can be drawn between our study findings and existing research about chronic non-cancer pain. For example, previous findings show that chronic pain patients do not always feel believed or validated by HCPs, their families, and friends (Nielsen, 2010; De Ruddere et al., 2016;De Ruddere and Craig, 2016; Nicola et al., 2019). Patients perceive others are convinced the pain is exaggerated or imagined (De Ruddere et al., 2014). Additionally, HCPs claim that the patient is not putting in enough effort or is not following their recommendations (Nicola et al., 2019).

These beliefs and prejudices can influence the behaviour of others in interaction with the survivor. For example, their environment ignores pain complaints, expresses less sympathy, and is less willing to help (De Ruddere et al., 2016). When prejudices result in devaluing, discrediting, or discriminating reactions towards the BCS, the survivor might feel stigmatized (Nielsen, 2010; Pescosolido and Martin, 2015; De Ruddere and Craig, 2016). Stigmatisation is a social process and manifests itself through mechanisms on an intrapersonal, interpersonal, and sociocultural level (Chaudoir et al., 2013; Pescosolido and Martin, 2015). The question remains how stigma can influence a person’s way of coping with pain. Because of experienced stigma, some patients feel guilty and consider themselves a burden to others. In accordance with our study findings, they try to deny and ignore the pain (De Ruddere et al., 2016; Nicola et al., 2019). Additionally, BCSs perceive that the media portrays cancer as a success story from which you can benefit as a person. Because of this positivity in awareness campaigns and fundraisings, BCSs feel pushed to think and react in a certain – socially acceptable – way and ignore their problems caused by cancer (Trusson and Pilnick, 2017). Some patients also apply others’ beliefs about the stigmatized characteristic to themselves, i.e. internalised stigma (Earnshaw and Quinn, 2012). For example, they doubt the credibility of their own pain complaints, lose their self-confidence, disregard the impact of pain on their lives, play down the pain, and as a result avoid pain talk (De Ruddere and Craig, 2016; Nicola et al., 2019). Others expect to experience prejudice by others in the future, i.e. anticipated stigma (Earnshaw and Quinn, 2012). For example, when patients experience a lack of empathy in HCPs, they feel isolated, rejected (De Ruddere et al., 2016), and will no longer express their pain symptoms or seek support from HCPs (Earnshaw and Quinn, 2012; Edlund et al., 2017; Granek et al., 2019; Nicola et al., 2019; Stangl et al., 2019). In contrast to this, our study results showed that a patient’s reaction to experiencing prejudices does not always result in a reduction of problem-solving behaviour. In some BCSs prejudices awaken a ‘fighting spirit’. It motivates BCSs to express pain until they feel heard by HCPs and their pain complaints are solved.

Moreover, when people experience stigma and their beliefs are challenged, they might perceive injustice (Scott et al., 2019; Penn et al., 2020). In the context of pain, perceived injustice refers to the perception that pain is not understood by others, feeling a sense of unfairness, and blaming others for their suffering (Lynch et al., 2022). In our study, a few BCSs blame other survivors for faking the pain or complaining too much to HCPs. These BCSs presume they cannot talk about pain themselves because this makes them an exaggerator or complainer. In contrast to this, some survivors do not blame others but blame themselves. They believe it is their responsibility to talk about their pain complaints and search for solutions when they need them. This is of great importance because research suggests that when a person blames others for their suffering, their acceptance of pain can be impeded (Carriere et al., 2018).

Not every cancer survivor is able to accept the changes that cancer imposes (Zebrack, 2000). Still, we did find some survivors that are able to accept and live with the pain. Pain acceptance implies that an individual continues to pursue life goals and valued activities despite pain, and stops trying to control or avoid pain experiences (Carriere et al., 2018). Feeling validated by others aids acceptance of pain, poses a barrier to internalised stigma, and increases problem-solving behaviour, which in turn can result in better functionality and increased quality of life (Nicola et al., 2019; Penn et al., 2019; Andrade Carvalho et al., 2021). Our study also indicates that experienced validation can stimulate problem-solving behaviour and acceptance of pain. For example, when HCPs acknowledge the pain complaints and react understanding, BCSs feel hopeful and encouraged to search for solutions. Feelings of frustration and anger are also reduced, facilitating acceptance of pain. However, the role of family and friends was less mentioned by our participants when talking about solving or accepting pain, in comparison with ignoring the pain. A possible explanation might be that a partner’s or friend’s responsiveness and solicitousness (e.g. providing pain medication, helping with chores, asking how they can help) are not necessarily regarded as validation (Andrade Carvalho et al., 2021). An important aspect of active engagement is expressing a shared responsibility in coping with pain and other complaints. For example, when a survivor’s partner engages in ‘we’ talk regarding coping with complaints, the survivor might be more inclined to look for solutions (Kraemer et al., 2011).

### 4.1. Limitations and strengths

Participant recruitment and data collection were organised online. As a result, BCSs with poorer digital literacy might have been less inclined to participate in this study. Furthermore, individuals who are willing to discuss experiences with others during a focus group, are presumably more assertive. Therefore, our results regarding coping can be biased. However, by combining an asynchronous and synchronous part for each focus group, we experienced that most participants felt safe expressing their thoughts. We noticed that some participants were more active during the written part and others more during the oral part, depending on their personal preferences. Another advantage of combining methods was that participants already got to know each other’s backgrounds in the asynchronous part. So there was no familiarisation necessary during the synchronous conversation. Participants felt immediately at ease and understood by their peers. Previous answers were explored in more detail in the synchronous focus groups, which also resulted in discussing new topics. Furthermore, participants had some time during the two parts to further reflect on the questions and answers given. Still, we need to be aware that by reading the answers of others, there could be the risk that participants were steered in a certain direction.

Furthermore, due to the limited study sample, caution is warranted regarding the generalisation of study results. Additionally, an unequal distribution was noted between the number of participants with acute versus chronic pain. We experienced that it was more difficult to find survivors who are willing to participate in research within the first 3 months after completing cancer treatment. Overall, similar coping patterns were identified between survivors with acute and chronic pain with only a slight difference in underlying motives or cognitions.

Finally, all participants were Caucasian. This poses a limitation to our study as the literature states the impact of racial/ethnic biases on pain assessment and treatment (Hoffman et al., 2016; Aelbrecht and De Maesschalck, 2019; Gehlert et al., 2021). In future research, it is necessary to undertake additional measures to ensure diversity in sampling.

## 5. Conclusion

BCSs often experience that pain is not timely assessed or adequately monitored during follow-up care. But how survivors deal with pain complaints and unmet needs varies. Distinct coping patterns were identified, i.e. ignoring pain, solving pain, and accepting pain. Predicting which coping pattern a person will tend to is not always straightforward. Intrapersonal but also interpersonal and societal factors play a role. Therefore, coping should not be seen as a stable characteristic but rather as a dynamic process. Additionally, it is of great importance that HCPs and the environment are aware of their influence on a survivor’s way of coping. To sufficiently address the problem of pain amongst cancer survivors, we need actions that tackle the health care system and its stakeholders, as well as the public debate concerning cancer follow-up care.

## Data availability statement

The raw data supporting the conclusions of this article will be made available by the authors, without undue reservation.

## Ethics statement

The studies involving human participants were reviewed and approved by the Committee for Medical Ethics affiliated with Ghent University Hospital. The patients/participants provided their written informed consent to participate in this study.

## Author contributions

YS: conceptualization, data curation, formal analysis, investigation, methodology, project administration, and writing–original draft. EL and PP: conceptualization, formal analysis, investigation, methodology, supervision, and writing–review and editing. All authors contributed to the article and approved the submitted version.

## Funding

Kom op tegen Kanker aided this research financially (grant number: KOTK/2020/11886).

## Conflict of interest

The authors declare that the research was conducted in the absence of any commercial or financial relationships that could be construed as a potential conflict of interest.

## Publisher’s note

All claims expressed in this article are solely those of the authors and do not necessarily represent those of their affiliated organizations, or those of the publisher, the editors and the reviewers. Any product that may be evaluated in this article, or claim that may be made by its manufacturer, is not guaranteed or endorsed by the publisher.

## Footnotes

^1^ www.focusgroupit.com
