# Supplementary material for: Survivors’ perceptions regarding the follow-up of pain complaints after breast cancer treatment: Distinct coping patterns
Source: Front Psychol. 2023 Jan 12;13:1063705. doi: 10.3389/fpsyg.2022.1063705 (PMC9879359; doi:10.3389/fpsyg.2022.1063705)
Supplement: Supplementary file 1 [file Data_Sheet_1.PDF]

Supplementary File 1: Topic guide for asynchronous focus groups

Question 1

Which pain problems do you experience?

Probes

- How did your pain problems arise?
- How long have you been experiencing pain problems?
- How have your pain problems evolved?
- How do you feel about these pain problems?
- What impact or effect does pain have on you?
- How do you feel about this impact or effect?
- How does pain affect your well-being?
- Does pain change the way you see yourself? How?
- How does pain affect your daily life?

Question 2

To what extent do you seek help for your pain problems? *This may involve visits to doctors and medical specialists, but also to physiotherapists, psychologists and other disciplines who offer support for your pain problems.*

Probes

- How do you approach seeking help?
- Where do you look for help?
- Who do you turn to for help?
- When do you seek help?

Question 3

What makes you seek help for your pain problems?

Probes

- Do you encounter obstacles in seeking help? What obstacles do you encounter?
- Are there things in your environment that make it less likely that you would seek help for your pain problems?
- Are there things in your environment that make it more likely that you would seek help for your pain problems?
- What would make it easier for you to seek help?

Question 4

To what extent have your pain problems been discussed by healthcare providers?

Probes

- How did healthcare providers discuss your pain problems?
- Were there certain things that made your pain problems open to discussion?
- What thresholds can you think of that prevented your pain problems from being discussed?
- What recommendations do you have for healthcare providers?

Question 5

How do you experience the care you are currently receiving for your pain problems?

Probes

- Do you feel you are getting the support you need?
- Do you feel that all necessary professionals are involved in your care? Who is missing?
- Do healthcare providers respond sufficiently to your pain problems? Why not?
- What do you think are shortcomings in the care of pain problems?

Question 6

Can you tell me more about how your close environment responds to your pain problems?

Probes

- Do you get the support and understanding you need and/or ask for? How do you feel about this?
- Is there a difference in how your close environment (for example family and friends) and healthcare providers show understanding for your pain problems? How does this difference occur?
- Do you feel that the level of support or understanding of your environment affects the way you cope with pain and pain treatment? How exactly?

Question 7

What do you feel is the general response of society towards pain after cancer treatment? *Think of understanding reactions that you notice, or possibly negative signals.*

Probes

- Can you give an example?
- How do you feel about this?
- Do you feel there are prejudices about pain after cancer? If so, which ones?
- Do you feel that this public response affects the way you deal with the pain and pain treatment? How exactly?

Question 8

Finally, the aim of this research is to develop stepped care for cancer survivors with pain problems. "Stepped care is a way to determine the amount and sequence of care services to provide each survivor with the necessary level of care. Each survivor is offered tailor-made

care depending on their needs and risk factors. Every survivor does not necessarily receive the whole package of services. We start with low-intensity care services and when necessary gradually adapt to more intense and complex care services." When you keep your own situation in mind, do you think the approach of stepped care might be useful during follow-up?

Probes

- Do you think there is still something missing in this approach?
- What role can you play in stepped care?
- How would you fulfill this role?
- When should stepped care start during follow-up?
- Where should stepped care start during follow-up?
